# Neural contrast sensitivity is not affected by myopic blur

**DOI:** 10.1038/s41598-025-15911-y

**Published:** 2025-08-20

**Authors:** Niklas Domdei, Jonas Müller, Lisa Renner, Julius Ameln, Katharina Breher, Wolf Harmening, Siegfried Wahl

**Affiliations:** 1 Carl Zeiss Vision International GmbH, Aalen, Germany; 2 Institute for Ophthalmic Research, Eberhard Karls University Tübingen, Tübingen, Germany; 3 Hochschule Aalen, Aalen, Germany; 4 University Eye Hospital Bonn, Rheinische Friedrich-Wilhelms-Universität Bonn, Bonn, Germany

**Keywords:** Myopia, Under-correction, Interference fringes, Neural contrast sensitivity, Psychophysics, Cone density, Albinism, Sensory processing, Quality of life, Refractive errors, Perception

## Abstract

The prevalence of myopia is increasing worldwide, accompanied by an increase of potentially under-corrected myopes. Because neural pathways are prone to adaptation in relation to the sensory input, we wondered to what extent neural contrast sensitivity (NCS) is altered in the presence of image blur arising from under-correction. No significant differences in NCS were observed between the three groups but were significantly equivalent between emmetropes, well-corrected, and under-corrected myopes (all *p* < 0.001). Across all healthy participants, NCS variability increased with increasing spatial frequency which could not be explained by variations in foveolar cone density. For an albinism patient, a large difference between the cut-off frequency and central cone density derived Nyquist sampling limit was observed. In conclusion, NCS is unaffected by myopia and remains stable for under-correction of up to 1.5 D. Furthermore, NCS testing can relate visual deficits to an underlying neurological disorder.

**Supplementary Information:**

The online version contains supplementary material available at 10.1038/s41598-025-15911-y.

## Introduction

Myopia, a common eye condition in humans with increasing prevalence worldwide^1,2^, leads to a blurred retinal image if inadequately corrected. According to recent studies, about 10 to 2% of the adult population is under-corrected^3–5^. Approximately 90% of all under-corrected myopes improve by 2 or 3 lines in visual acuity when fully corrected^6^, which converts into an estimated refractive error of about 0.50 to 0.75 D^7,8^. Due to the increasing cases of myopia, it can be assumed that the number of under-corrected eyes has also grown and will be growing even further in the future. Additionally, purposefully induced under-correction is also discussed as a possible strategy to slow myopia^9,10^.

Spatial vision, and specifically, our ability to detect visual contrast, is a product of optical and neural processes that start with a retinal image in the eye and ultimately lead to visual perception. The optical properties of the eye that determine the image quality formed on the retina^11,12^can be mathematically described by the modulation transfer function (MTF). On the neural end, the neural transfer function (NTF) describes the processing of that image by the retina and ascending visual pathways^13^. The psychophysically measurable ability to detect contrast, the contrast sensitivity function (CSF), can thus be written as,

$$\:CSF=MTF\:\cdot\:\:NTF\:$$^14,15^.

Direct measurement of the CSF is achieved by testing the minimum visible contrast at varying spatial frequencies^16^. However, if a patient’s CSF deviates from the basic healthy CSF, it is often unclear whether this is the result of impaired optics (MTF) or neural pathways (NTF). Furthermore, it was observed that a deterioration of the eye’s optical components is followed by a degradation of the neural components, according to the use-it-or-lose-it principle^17^.

By using adaptive optics to eliminate any MTF related influences^18–20^one specific component of the NTF, the neural contrast sensitivity (NCS), could be measured directly. This was done, for example, with patients suffering from keratoconus, which is characterized by an extremely blurry retinal image due to highly irregular corneal aberrations. These patients were unable to achieve normal NCS^21^. It was argued that due to the unusually high optical aberrations of their eyes the visibility particularly of high spatial frequencies is significantly reduced. In turn, the missing input power of these frequencies lead to a degradation of the processing channels sensitive to high spatial frequencies.

Direct measurement of the NCS can also be achieved with an interferometric approach that focusses two coherent light beams into the eye’s pupil, producing an interference fringes stimulus on the retina^22–24^. In this so called Maxwellian view configuration^25^the eye’s optics are bypassed and an aberration-free presentation of the stimulus is achieved. Such a system could furthermore be beneficial to assess the underlying reason of poor visual performance. For example, if significantly reduced NCS values are observed for a patient, it can be assumed that vision is limited due to a neurological or retinal disorder instead of optical deficiencies.

Myopia as a refractive error can be corrected with for example spectacle lenses, which allows well-corrected young myopes to see clearly at all distances. Under-correction of this refractive error leads to a blurry image when looking into the distance. For people doing mainly near-work throughout the day, like working in an office or craftspeople, this condition is less of a problem. But people with less near work, for example, students who attend classes in lecture halls, are exposed to longer periods of blurry vision if left un- or under-corrected. In these cases, long-term adaptation to blur could result in degraded NCS, like it was observed for keratoconus patients. On the contrary, it is hypothesized that myopia is the result of an abnormally high contrast signaling in the retina, caused by mutations in the cone opsin, which in turn interferes with the emmetropization process^26^. Here, we tested whether NCS in myopic eyes is altered due to blur. For this a spatial-light-modulator-based interferometer instrument was used allowing for an aberration free stimulation to study the NCS of emmetropes, well-corrected, and under-corrected myopes. Additionally, the resulting eye-healthy NCS is compared with the NCS of an albinism patient with known congenital foveal hypoplasia, thereby probing the method’s capability to detect an estimated abnormal NCS.

## Results

Neural contrast sensitivity (NCS) was psychophysically assessed in the dominant eyes of a total of 48 participants by projection of interference fringes, a method which is known not to be affected by individual optical errors. Participants were assigned into three different groups based on the participant’s currently needed, determined via subjective refraction, and available optical correction (Table 1). The first group consisted of emmetropes (control) defined by an objectively measured spherical equivalent of ≥ − 0.5 D (*N* = 11)^27^. The second group were the well-corrected myopes (*N* = 26) defined by a maximum difference of < 0.5 D (sphere) between needed and available correction. The third group, the under-corrected myopes (*N* = 11), was defined by an offset of ≥ 0.5 D (sphere) between needed and available correction, and an improvement in visual acuity by at least one line on the visual acuity chart when best corrected. Seven of the eleven under-corrected myopes never underwent a vision test, the other four had their last refraction several years ago (self-reported).

**Table 1:** Demographics, dominant eye’s refraction and visual acuity for all participants sorted by study groups. Axial length (in mm) was measured with IOL master 700 (Carl zeiss meditec, Dublin, CA, USA). Objective refraction (stated as spherical equivalent in Diopter) was measured with iProfiler plus (Carl zeiss vision gmbh, Aalen, Germany). Subjective refraction was determined using a phoropter (ZEISS Visuphor 500, Carl zeiss vision gmbh, Aalen, Germany) and screen (ZEISS Visuscreen 500, Carl zeiss vision gmbh, Aalen, Germany) with letter optotypes at 4.5 m distance. The current prescribtion was assessed by measuring the lenses of the worn spectacle with a focimeter (ZEISS Visulens 500, Carl zeiss vision gmbh, Aalen, Germany). BCVA, Best-Corrected Visual Acuity; Cyl, Cylinder; D, Diopter; LogMAR, Logarithmic Minimum Angle of Resolution; SE, Spherical Equivalent; Sph, Sphere.

ID	Sex	Age	Axial length (mm)	Objective refraction (SE in Diopter)	Subjective refraction (D)		Spectacle correction (D)		Resulting under-correction SE (D)	Acuity with current correction (LogMAR)	BCVA (LogMAR)
					Sph	Cyl	Sph	Cyl			
Emmetrop_001	m	24	23.9	− 0.42	–	–	None		–	− 0.2	− 0.2
Emmetrop_002	f	23	22.7	− 0.45	–	–	None		–	− 0.2	− 0.2
Emmetrop_003	m	25	22.6	0.09	–	–	None		–	− 0.2	− 0.2
Emmetrop_004	f	24	24.0	− 0.18	–	–	None		–	− 0.2	− 0.2
Emmetrop_005	f	29	22.7	0.36	–	–	None		–	− 0.2	− 0.2
Emmetrop_006	m	30	22.6	− 0.27	–	–	None		–	− 0.2	− 0.2
Emmetrop_007	f	33	23.7	− 0.49	–	–	None		–	− 0.2	− 0.2
Emmetrop_008	f	21	23.6	− 0.38	–	–	None		–	− 0.2	− 0.2
Emmetrop_009	f	28	25.3	0.38	–	–	None		–	− 0.2	− 0.2
Emmetrop_010	f	34	23.9	− 0.16	–	–	None		–	− 0.2	− 0.2
Emmetrop_011	m	39	22.1	0.31	–	–	None		–	− 0.2	− 0.2
MyoWell_001	f	29	24.7	− 4.45	− 3.50	− 1.50	− 4.00	− 1.50	0.50	− 0.2	− 0.2
MyoWell_002	m	33	24.1	− 2.30	− 1.75	− 1.75	− 1.75	− 1.00	− 0.38	− 0.2	− 0.2
MyoWell_003	f	23	23.1	− 1.98	− 1.75	− 0.25	− 2.00	− 0.25	0.25	− 0,2	− 0.2
MyoWell_004	f	19	25.2	− 1.96	− 1.25	− 0.50	− 1.00	− 0.25	− 0.38	− 0.2	− 0.2
MyoWell_005	f	31	24.1	− 2.19	− 1.75	− 0.50	− 1.50	− 0.50	− 0.25	− 0.1	− 0.2
MyoWell_006	m	20	24.1	− 2.14	− 1.50	− 1.75	− 1.50	− 1.25	− 0.25	− 0.2	− 0.2
MyoWell_007	f	32	23.2	− 2.09	− 1.25	− 1.50	− 1.25	− 1.50	0	− 0.2	− 0.2
MyoWell_008	f	22	24.0	− 1.98	− 1.25	− 0.25	− 1.00	− 0.25	− 0.25	− 0.2	− 0.1
MyoWell_009	f	25	23.7	− 1.76	− 1.75	− 0.25	− 1.50	− 0.25	− 0.25	− 0.2	− 0.2
MyoWell_010	m	27	23.7	− 2.13	− 1.75	0	− 1.50	0	− 0.25	− 0.1	− 0.2
MyoWell_011	m	28	26.5	− 4.08	− 4.00	0	− 4.00	0	0	− 0.2	− 0.2
MyoWell_012	f	35	23.9	− 2.15	− 1.75	− 0.5	− 1.50	− 0.50	− 0.25	− 0.2	− 0.2
MyoWell_013	f	23	24.2	− 0.85	− 0.25	− 0.75	− 0.75	− 0.50	0.38	− 0.1	− 0.1
MyoWell_014	m	37	25.1	− 2.01	− 1.50	− 0.50	− 1.75	− 0.50	0.25	− 0.1	− 0.2
MyoWell_015	m	22	23.7	− 1.94	− 1.50	− 1.00	− 1.50	− 0.75	− 0.13	− 0.2	− 0.2
MyoWell_016	f	20	22.7	− 1.36	− 1.50	− 0.50	− 1.25	− 0.50	− 0.25	− 0.2	− 0.1
MyoWell_017	f	23	24.2	− 1.94	− 1.50	− 0.50	− 1.25	− 0.50	− 0.25	− 0.2	− 0.1
MyoWell_018	m	22	23.2	− 1.56	− 1.50	0	− 1.75	0	0.25	− 0.2	− 0.2
MyoWell_019	f	39	24.6	− 2.72	− 2.50	− 0.75	− 2.75	− 0.75	0.25	− 0.2	− 0.2
MyoWell_020	m	24	24.0	− 4.92	− 4.00	− 0.75	− 4.00	− 0.50	− 0.13	− 0.2	− 0.2
MyoWell_021	m	30	24.0	− 2.44	− 1.75	− 1.75	− 1.75	− 1.75	0	− 0.2	− 0.2
MyoWell_022	m	29	26.5	− 4.14	− 3.75	0	− 3.75	0	0	− 0.2	− 0.2
MyoWell_023	f	29	24.7	− 3.40	− − 2.75	0	− 2.75	− 0.25	0.13	− 0.2	− 0.2
MyoWell_024	f	25	24.3	− 2.71	− 1.75	− 0.50	− 1.50	− 0.50	− 0.25	− 0.2	− 0.2
MyoWell_025	m	36	24.9	− 2.13	− 1.25	− 1.25	− 1.25	− 1.25	0	− 0.2	− 0.2
MyoWell_026	f	34	24.4	− 6.65	− 6.00	− 0.75	− 6.00	− 0.75	0	− 0.2	− 0.2
MyoUnder_001	m	31	23.9	− 0.83	− 0.50	− 0.75	None		− 0.88	0.0	− 0.2
MyoUnder_002	m	33	23.5	− 0.54	− 0.50	− 1.00	None		− 1.00	0.1	− 0.1
MyoUnder_003	f	20	23.8	− 1.02	− 0.50	− 0.75	None		− 0.88	0.1	− 0.2
MyoUnder_004	f	20	23.5	− 0.80	− 0.75	− 0.25	− 0.25	− 0.25	− 0.50	0	− 0.2
MyoUnder_005	f	35	23.8	− 1.10	− 0.75	− 0.75	None		− 1.13	0.1	− 0.2
MyoUnder_006	f	25	23.4	− 0.49	− 0.50	− 0.25	None		− 0.63	0	− 0.2
MyoUnder_007	f	23	23.3	− 2.95	− 2.50	− 1.00	− 2.00	− 0.50	− 0.75	0	− 0.1
MyoUnder_008	m	23	23.7	− 1.49	− 1.00	− 0.25	None		− 1.13	0.1	− 0.2
MyoUnder_009	m	22	26.2	− 3.00	− 3.00	0	− 1.50	0	− 1.50	0.2	− 0.2
MyoUnder_010	m	20	24.4	− 0.50	− 0.75	− 0.25	None		− 0.88	0.1	− 0.2
MyoUnder_011	m	27	26.9	− 7.49	− 7.25	− 1.00	− 6.50	− 0.75	− 0.88	− 0.1	− 0.2
Albinism_001	f	31	21.3	0.94	2.50	− 2.25	2.50	− 2.25	0	0.2	0.2

### Emmetropes and myopes have equal neural contrast sensitivity

**Fig. 1:**
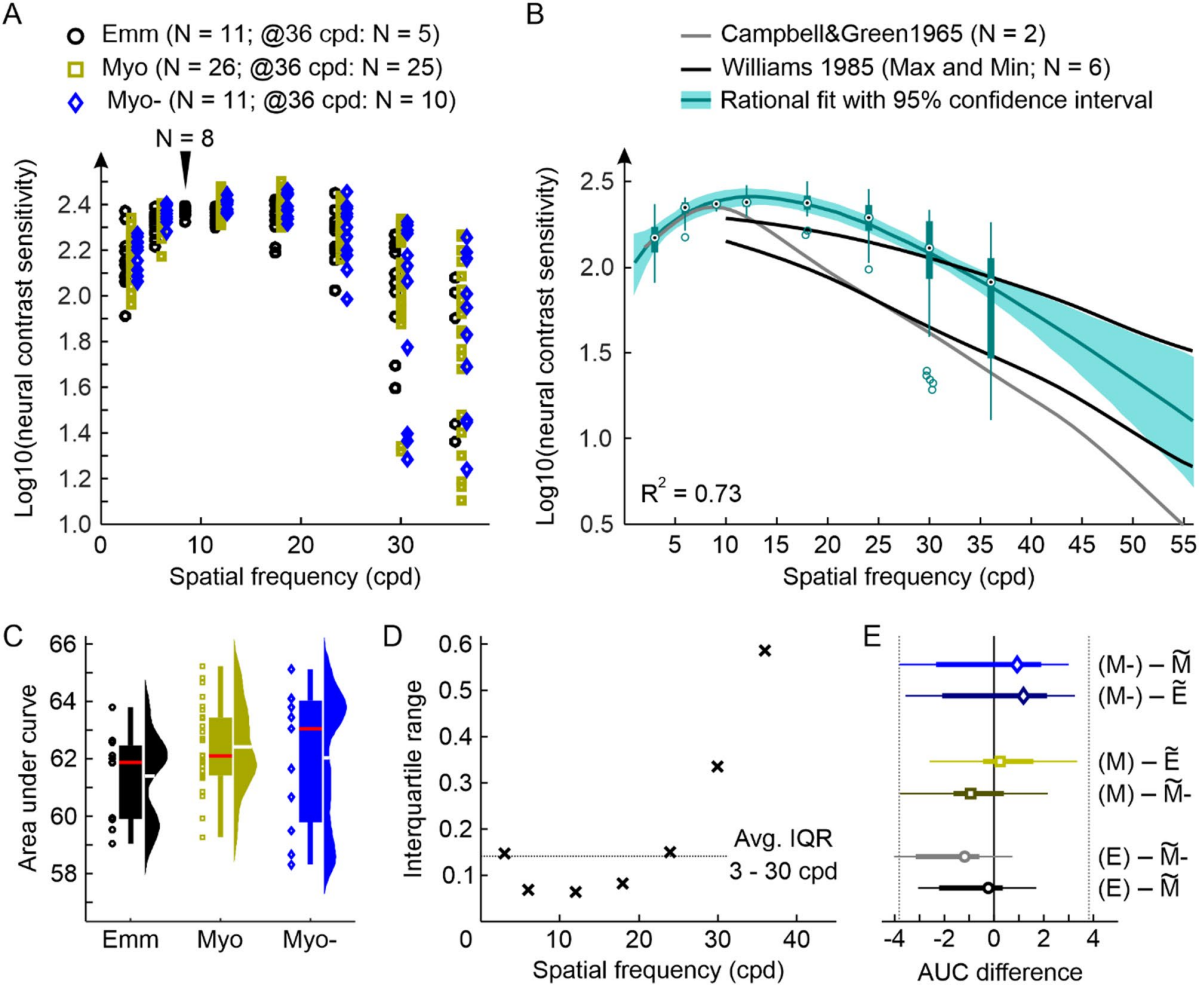
Neural contrast sensitivity measured with interference fringes and analysis. (**A**) Log10(NCS) for the three different groups tested. Marker position states each participant’s median NCS of three repeated measurements. Not all participants were able to perceive the 36-cpd-stimulus. The resulting number of valid measurements for this spatial frequency is stated in the figure legend. (**B**) Based on the observation that NCS is similar across the different groups (see (**C**–**E**) for the analysis) data was pooled (plotted as compact boxplot with circles marking numerical outliers) and fitted with a rational function ((ax^2^ + bx + c)/(x + d)) including all data points. For comparison, literature data from Campbell&Green^22^and Williams^30^ is provided. (**C**) Area under the NCS curve from 3 to 30 cpd. White lines indicate the mean, red lines the median value. Differences between groups are not significant (Mann-Whitney U-test, all *p* > 0.05). (**D**) Determination of the average interquartile range per test SF from pooled thresholds for 3 to 30 cpd. (**E**) Test for equivalence using the average IQR per tested SF as lower and upper limit (l1 = − 3.81; l2 = 3.81) reveals a significant equivalence between AUCs of the three groups (TOST, all *p* ≤ 0.01). The thick horizontal lines display the group’s interquartile range (25–75%) with the thin lines representing the whiskers. The group’s median is given by the marker. Emmetrope: Emm, E; Myope (well): Myo, M; Myope (under): Myo-, M-; $\:\stackrel{\sim}{X}=group\:median$.

Median NCS values-in the following reported as log10 units - were similar across all three groups (see Fig. 1A). Sensitivity increased from a spatial frequency (SF) of 3 cycles per degree (cpd) (2.13, 2.17, and 2.20; for emmetropes, well-corrected, and under-corrected myopes, respectively), over 6 cpd (2.32, 2.35, and 2.37), to 12 cpd (2.36, 2.40, and 2.40). Because peak NCS was reported earlier at about 10 cpd, a subset of emmetropic participants (*N* = 8) was tested at 9 cpd. For this subset, median NCS measurements were equal with their respective median NCS at 12 cpd (both 2.36). The group of under-corrected myopes reached the maximum median NCS at 18 cpd (2.35, 2.38, and 2.37). Beyond this SF, NCS slowly decreased for all three groups, measured at 24 cpd (2.21, 2.29, and 2.30), 30 cpd (2.10, 2.11, and 2.13), and 36 cpd (1.90, 1.92, and 1.89). Overall variability within the three groups was very low for SFs ≤ 24 cpd, given average interquartile ranges for individual NCS values of 0.09, 0.08, and 0.11. The smallest interquartile range was observed for NCS measurements at 12 cpd with 0.06, 0.08, and 0.05, and the highest at 36 cpd with 0.61, 0.41, and 0.71 (for emmetropes, well-corrected, and under-corrected myopes, respectively). Several participants were not able to perceive the 36 cpd interference fringes even at the highest contrast setting (*N* = 6 (55%), 1 (4%), and 1 (9%)) making a threshold determination impossible. Based on the observation of similar NCS values, the measurements were pooled across all participants (*N* = 48) to calculate the normal NCS curve via a Rational-2-1 fit, describing the NCS of young (20–39 years old) and healthy humans (Fig. 1B).

For a statistical comparison of the recorded NCS functions, the area under the curve (AUC) was calculated for SFs between 3 and 30 cpd, neglecting 36 cpd values due to the very high variability and incompleteness of the data available (Fig. 1C). The median AUC value for the emmetrope group was 61.87 [Interquartile Range: 59.92 to 62.35]. In the two myopic groups, the median AUC was 62.12 [61.47 to 63.44] for the well-corrected and 63.06 [60.08 to 63.95] for the under-corrected participants. The observed small differences between the three groups were not significant (all *p* > 0.1; Mann–Whitney U-test). In addition, similarity of the three groups was statistically assessed by equivalence testing following the two one-sided tests approach (TOST)^28,29^. For the TOST, it is necessary to define the limits within which any observed fluctuations are tolerable. Usually, this limit is defined in relation to the standard deviation (Cohen’s d). Because the data here was not normally distributed the required limit was instead determined based on the average interquartile range of 0.14 of NCS measurements across all participants at spatial frequencies between 3 and 30 cpd (Fig. 1D). Given this observed variability range for NCS measurements, the AUC would change by ± 3.82, which was then used as the test boundaries with L1 = − 3.82 as the minimum and L2 = 3.82 maximum limit. With this limit definition, the three groups showed significantly equivalent populations of AUC for the measured NCS curves (all *p* ≤ 0.001, see Fig. 1E).

### Neural contrast sensitivity is independent of refractive error and age

**Fig. 2:**
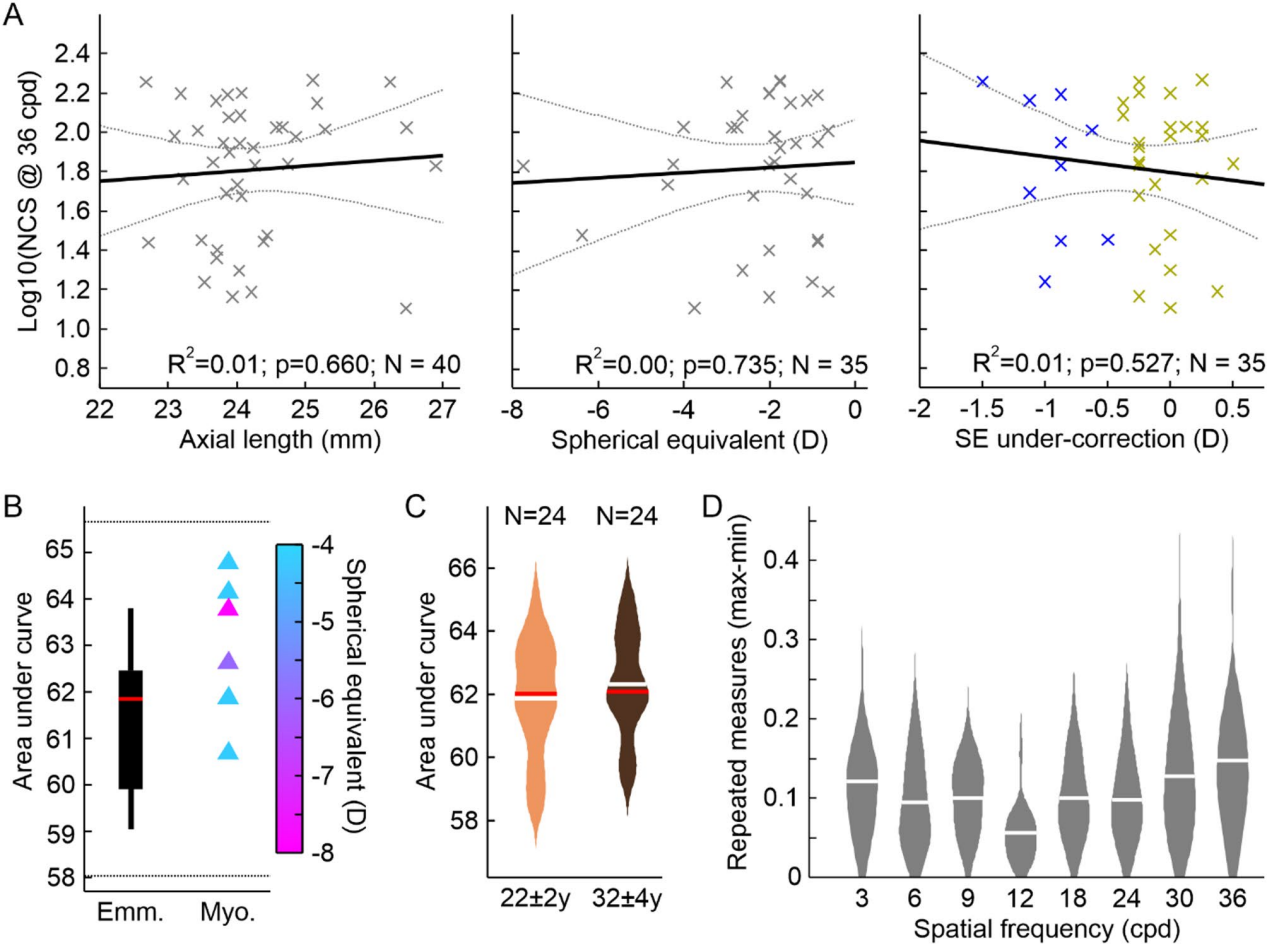
Neural contrast sensitivity is neither correlated with the eye’s refraction nor age. (**A**) None of the tested correlations between observed NCS at 36 cpd and physical parameters of the eye (axial length, refraction given by spherical equivalent, or under-correction) was significant. For axial length NCS measurements were pooled across all three groups, for spherical equivalent and under-correction NCS measurements were pooled from both myopic groups. Grey dotted lines indicate 95% confidence intervals. (**B**) The AUC comparison between emmetropes (SE > − 0.5 D; *N* = 11) and myopes with SE ≤ − 4 D (*N* = 6) revealed a non-significant difference (*p* = 0.06), but significant equivalent NCSF (*p* = 0.02). TOST limits indicated by the dashed lines. (**C**) Division of the pooled data set into two age groups (22 ± 2 years and 32 ± 4 years) showed no significant difference (*p* = 0.48), but significant equivalence (*p* < 0.001). (**D**) Low variability for repeated measurements: 93% of repeated NCS measurements at a given spatial frequency showed a maximum difference of 0.2 log10 NCS or less between the highest and smallest value recorded. White lines indicate the mean, red lines the median value.

A noteworthy observation was the increasing variability for NCS measurements at high SF (≥ 30 cpd), which was similarly high for all three groups at 36 cpd (Fig. 1A). To find an explanation for this, the individual’s median NCS at 36 cpd of all groups was tested for correlation with axial length (Fig. 2A), without revealing any significance (*p* = 0.48). Additionally, using the myopic eyes’ data only, the spherical equivalent and the amount of under-correction were compared with the respective individuals’ NCS at 36 cpd. Again, both correlations were not significant (both *p* > 0.5).

Based on this observation, the NCS curves of the six participants, showing the highest myopia of the cohort here (≤ − 4 D) were tested in comparison with the emmetropic group. There was no significant difference between the AUCs of the two groups (*p* = 0.06), instead both were significantly equivalent (*p* < 0.05), given the above-defined limits of equivalence (Fig. 2B).

Testing any age-related influence on the NCS within 20 and 40 years, the data set was divided into two subgroups. One group with an average age of 22 ± 2 years (*N* = 24) and the second (*N* = 24) with 32 ± 4 years (Fig. 2C). Statistical comparison of the two groups showed no significant difference, but significant equivalence (*p* = 0.50, and *p* < 0.001, respectively).

To assess the repeatability and therefore reliability of the method, the spread of the three repeated NCS measurements per SF was analyzed given by the difference between the maximum and minimum value (Fig. 2D). Overall, 95% of the repeated measurements had a spread of 0.21 log10(NCS). The average spread decreased from 0.12 (STD: ±0.09) at 3 cpd to its lowest value of 0.06 (± 0.04) at 12 cpd and increased for higher SFs up to 0.15 (± 0.10) at 36 cpd.

### Estimated cut-off frequency, Nyquist limit, and visual acuity

**Fig. 3:**
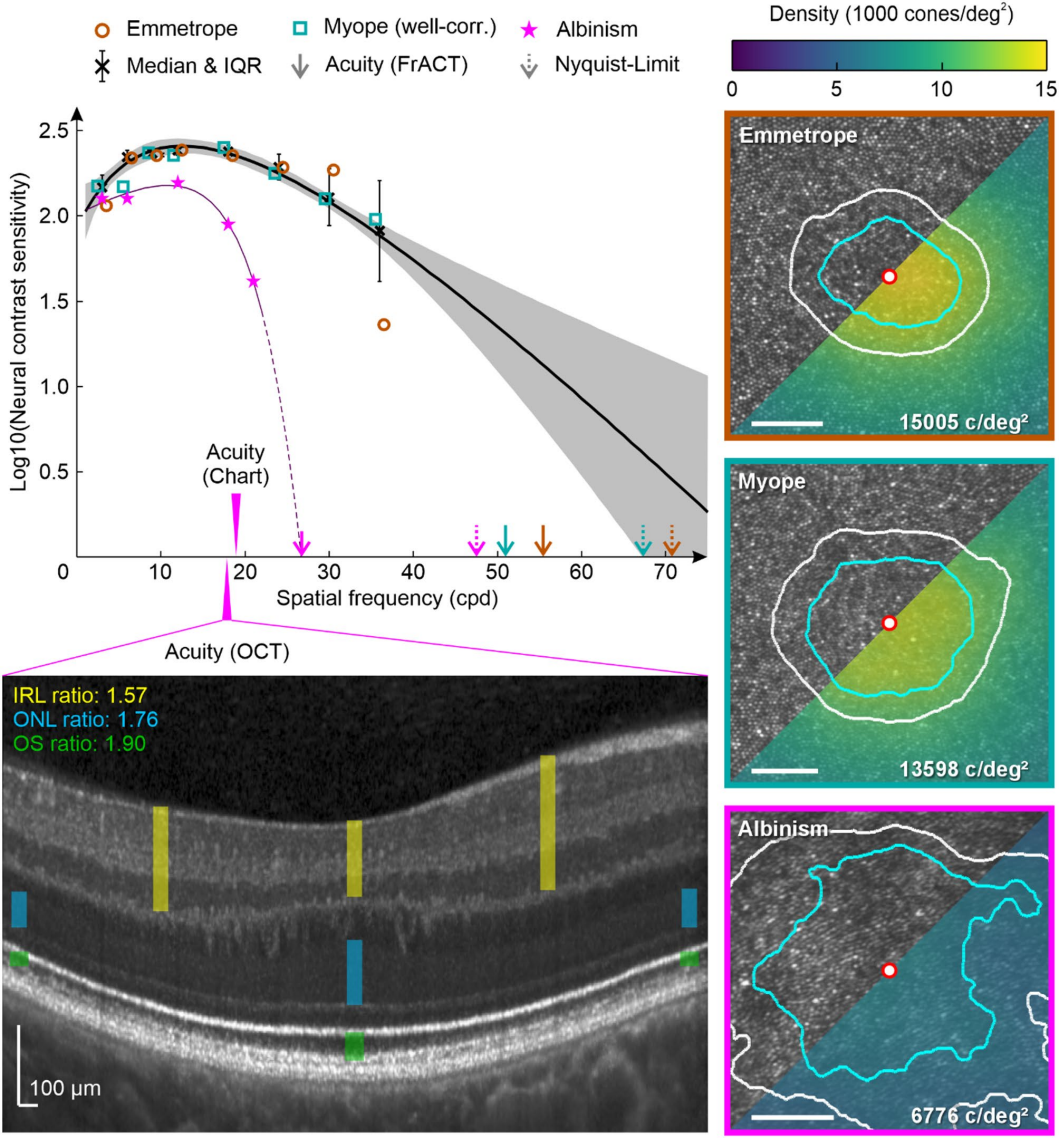
The general healthy neural contrast sensitivity curve and comparison with an albinism patient. At each tested spatial frequency all available NCS measurements were pooled and the resulting Medians (black Xs, error bars indicate the respective interquartile range) were fitted with a quadratic function (black line), the grey lines show the 95% confidence interval of the fit. Dotted lines indicate the extrapolation of the NCS curve for higher spatial frequencies not tested here. The estimated cut-off frequency (intersection of the fitting line with the abscissa) matches with the Nyquist limit of two study individuals. The Nyquist limit was obtained from foveolar cone densities based on AOSLO imaging (right side). The best-corrected visual acuity measured with FrACT for these two healthy individuals was slightly reduced compared to the Nyquist limit. For the albinism data a rational function of degree (1,2) yielded the best fit. The estimated cut-off frequency was closely related to the best-corrected visual acuity (FrACT), but significantly less than the AOSLO based Nyquist limit. Using the method recently proposed by Woertz et al. to predict the visual acuity from OCT images in albinism patients^31^. The predicted visual acuity for the tested albinism patient matches with the Snellen chart visual acuity but is considerably less than the estimated cut-off frequency and FrACT visual acuity. For the foveolar cone images and densities, the central marker denotes the cone density centroid and the contour lines are the 90% (cyan) and 80% (white) central cone contours. IRL: Inner retinal layers; ONL: Outer nuclear layer; OS: Outer segments.

Two participants were selected for an additional analysis for the following reasons: the NCS function of the myopic participant was closely aligned with the fit function, while the emmetropic participant had a very low NCS value at 36 cpd, which could be due to abnormalities in the foveolar cone mosaic after any refractive influence was ruled out (see Fig. 2A). The foveolar Nyquist limit of these two representatively analyzed participants was similar (emmetropic: 70.7 cpd, well-corrected myopic: 67.3 cpd) and slightly lower compared to the cut-off frequency based on the fit function for healthy participants (81 cpd) and within the 95% confidence interval of the fit (Fig. 3). The FrACT-derived cut-off frequency was lower than the Nyquist limit and with a comparable offset in both analyzed eyes (55.4 cpd and 51.0 cpd, for the emmetropic and the myopic participant, respectively).

### Neural contrast sensitivity for an albinism patient

The normal NCS curve from the pooled healthy data set (see above) was then compared to the NCS recording of an albinism patient with known foveal hypoplasia. Only for the lowest SF tested (3 cpd), the NCS measurements of the albinism patient overlapped with the normal curve. At a SF of 12 cpd, the NCS curve for the albinism patient reached its maximum, like the normal NCS function, but was significantly lower (albinism median NCS = 2.19; normal NCS = 2.41). While the normal NCS shows a rather flat decreasing slope after the peak, the albinism’s NCS decreases steeply, such that the NCS at a SF of 24 cpd could not be tested in our system, limited by the maximum available contrast of 6.3% (= log10(NCS) of 1.2).

In a final step, the predicted cut-off frequency for the albinism patient, estimated by fitting a Rational-1-2 function (R^2^ = 0.99), was compared to the Nyquist limit based on the individual foveolar cone mosaic (Fig. 3, lower right image). The predicted cut-off frequency from the NCS-fit (26.7 cpd) was much lower than the Nyquist limit of the foveola (47.5 cpd). But it matched exactly with the cut-off frequency obtained by converting the minimum angle of resolution from visual acuity testing. However, when using the minimum angle of resolution from a commonly used letter chart acuity test, this estimated cut-off frequency was lower (18.9 cpd) compared to the albinism NCS-fit prediction. A recently proposed method by Woertz et al. was applied to predict visual acuity based on structural features of the individual retina obtained via optical coherence tomography (OCT)^31^. This OCT-derived cut-off frequency (17.8 cpd) was again lower than the NCS-based estimate, but close to the letter chart acuity.

## Discussion

Motivated by a world-wide increasing prevalence of myopia and the associated increasing numbers of potentially uncorrected and under-corrected myopes, the aim was to assess whether neural contrast sensitivity (NCS) is affected and possibly reduced, due to long-term neural adaptation to myopic blur. Using an improved interferometric setup that enabled aberration-free stimulus presentation in a larger number of naïve participants compared to previous systems^22,23^, we found equivalent NCS functions for emmetropes, well-corrected myopes, and under-corrected myopes.

Previous studies using a similar approach with adaptive optics (AO) to correct optical errors, reported degraded NCS in keratoconus patients. This result was thought to be caused by long-term adaptation to optical errors^32^. Other studies reported orientation specific NCS functions for keratoconus patients^33^ as well as in cases of astigmatism^34^ that were correlated with the individual asymmetrical optical aberrations of their eye. For myopic participants, a decreased visual resolution after AO correction compared to emmetropic participants was reported^35,36^. A possible explanation was that such a reduction in sensitivity could result from retinal stretching^37^ in myopia leading to a reduced foveolar cone density^38^. Additionally, higher order aberrations, which cannot be corrected by lenses, show a tendency to be increased in myopia. This could lead to a degraded image quality in even well-corrected myopes compared to emmetropes^39^. The contrast detection task applied here revealed that NCS functions for emmetropes and well-corrected myopes were significantly equivalent. A specific comparison of the NCS functions between the six most myopic participants (≤ -4D) and emmetropes, also showed a significant equivalence. This is in line with the finding that foveal acuity, assessed via an interferometer, was similar in terms of angular units between corrected myopes and emmetropes^40^. Thus, if there are elevated higher order aberrations in myopes, these would only play a minor role for the overall image quality, even at high spatial frequencies. Furthermore, latest advances in retinal imaging made it possible to resolve the foveolar cone mosaic and it was reported that in fact, myopic and thus longer eyes have on average slightly higher angular foveal cone densities compared to shorter, emmetropic eyes^41,42^. The reason for the different observations from AO and interferometer based studies is most likely that the AO stimulus is affected by unknown residual uncorrected wavefront errors and retinal magnification, while the interferometer stimulus is largely independent of the eye’s aberrations and axial length^43^.

Based on the observations in keratoconus patients that show a decreased NCS assumed to be due to long-term adaptation processes, a diminished NCS could be expected for under-corrected myopes as well. However, contrary to this expectation, we found no NCS reduction, and instead NCS functions between emmetropes and long-term under-corrected myopes were significantly equivalent. The absence of any measurable effect could be explained simply by too short or too little exposure to under-correction. In fact, the duration and magnitude of under-correction tested here would be likely to have impacted NCS: seven of the eleven under-corrected myopes never had their refraction tested before, which means several years of myopic blur, and the remaining four had their last refraction several years ago (according to the participants). Considering an average change rate of 0.135 D per year for low progressing myopes between 12 and 22 years of age^44^, it can be assumed that at least 10 of the 11 under-corrected myopes were long-term under-corrected by about 0.5 D or more for a minimum of 6 months. At a far viewing point, an under-correction of 0.5 D, largely diminishes the modulation transfer function, leading to a significant signal reduction of medium spatial frequencies (SFs,10 to 20 cpd) and complete elimination of high SFs (≥ 30 cpd) (see Supplemental Fig. S1). The simulated reduction in contrast for a 12 cpd stimulus is about 0.5 log10 units for 0.5 D under-correction. That even small amounts of defocus have a measurable impact on vision is supported by the observation of a significant CS reduction at 16 cpd with 0.5 D blur in a psychophysical study^45^. In conclusion, neural mechanisms have to be at play to prevent a long-term degradation of the NCS with myopic defocus blur. While the extreme optical aberrations of eyes with keratoconus can never be fully corrected in daily life, myopic defocus is symmetric and can be easily corrected with lenses. Furthermore, under-corrected myopes have their far point close by, allowing them to see clearly at short distances. Thus, during near work, their high spatial frequency channels are likely to be sufficiently stimulated in under-corrected myopes and in consequence remain active. To note, inhibition of blur adaptation was shown to be present only if the exposure to clear vision exceeds the defocused periods^46^. Meaning that for an under-corrected myope any long-term adaptation effect from blurry distance viewing is counter-acted by the clear view during near work and successfully inhibited, when the time of near-work exceeds the time of distance viewing. A situation that is likely to be exhibited in normal daily activity of students as in the cohort of this study. On the contrary, in keratoconus the incomplete correction of the irregular aberrations results in sustained image blur. The resulting insufficient stimulation of the high spatial frequency channels, without any periods of clear vision to inhibit any long-term adaptation, ultimately leads to a permanent visual function loss.

Across all participants, the NCS peak was found at about 12 cpd (Fig. 1A,B), which is higher compared to previous studies reporting peak NCS at 8 cpd or 10 cpd^22,23^. Because in the original study protocol NCS was only tested at 6 and then 12 cpd, a small subset of emmetropic participants was tested at a SF of 9 cpd. For these participants equal NCS measurements at 9 and 12 cpd were observed. This supports, even though not specifically measured, the peak SF to be rather in between the two SFs. It can be furthermore estimated that the 12 cpd measurements are closer to the true peak sensitivity than the 6 cpd from the observation that for 12 cpd the standard deviation across the tested participants (Fig. 1D), as well as the spread for repeated measurements in the same participants (Fig. 2D), was lowest.

The absolute NCS values for low SFs (3 and 6 cpd) were similar to those reported by Campbell and Green^22^. In contrast, the observed NCS at high SFs (≥ 18 cpd) was higher than the previous reports, but with a similar slope for increasing SFs. Compared to Williams’^30^, the currently reported NCS values are slightly higher than the values reported for the most sensitive participants. A possible explanation for this could be that the center wavelength in our setup was 550 nm compared to 630 nm in the former studies. 550 nm light stimulates L- and M-cones equally, while 630 nm activates L-cones more than M-cones. It was shown that monochromatic NCS measurements at 543 nm are increased by about 0.15 log10 NCS compared to 623 nm^47^, which corresponds to the offset between measured and reported (Williams) NCS for SFs from 12 to 24 cpd.

The observation of similar NCS functions measured for such a large cohort contradicts earlier reports in which the NCS function was calculated based on the modulation transfer function and the CS function^48^. Such calculation led to highly variable and individual NCSFs, especially at medium SFs between 3 and 20 cpd. The problem of calculating the NCS function very likely arises from individual neural amplification of contrast under habitual optical aberrations^19,49^. Because this idiosyncratic neural gain is unknown when measuring the CS function, the calculated NCS function overestimates the real NCS function, which appears to be robust and similar across young healthy participants shown by the results in this study.

For high SF (≥ 30 cpd) a striking variability increase between individual NCS values was observed, with the interquartile range increasing from about 0.15 (≤ 24 cpd) to 0.6 at 36 cpd. Such idiosyncratic fluctuation of NCS at high SFs has been observed before with a difference of 0.4 log10 units between the highest and lowest sensitivity values^22,30^. The refractive state of the eye, axial length, and under-correction showed no correlation with NCS measurements at 36 cpd (or 30 cpd, data not shown), suggesting these factors are not the source of the observed effect (see also supplement Fig. 2).

It is known that even in the healthy population large variations of about 45% in the foveolar cone density (cone density ranges reported from Reiniger et al.^50^: 10,692 to 16,995 cones/deg^2^ or from Wang et al.^42^: 11,999 to 19,001 cones/deg^2^) occur. To test such effect on NCS the cone mosaics of two healthy participants were analyzed, for whom significantly varying NCS values at 36 cpd were measured. The central cone density for the emmetrope was higher (15,005 c/deg^2^) than for the well-corrected myope (13,598 c/deg^2^), but the NCS value at 36 cpd was lower (1.36) compared to the well-corrected myope (1.98) with less densely packed cones. This makes variations in the central cone density between participants rather unlikely to be the cause for the also in earlier studies observed increased variability at highly SFs^30^. Another possible explanation is a masking effect due to vitreous opacities or an instable tear film. For example, it was reported that a stable tear film increases visual acuity, while acuity decreases for dry eyes^51^. Although an interferometric system is supposed to bypass the eye’s optics, it is unclear to what extent the laser beams interfere with particles in the tear film, possibly degrading stimulus quality to an unknown extent. Participants were not specifically asked if they suffer from chronic dry eye. Artificial tear drops were offered during the ongoing experiment when a participant noticed any dry eye symptoms. Further research is needed with a carefully selected study cohort to directly test the impact of these hypothetical factors impacting interference fringe visibility.

Additionally to any blur adaptation leading to reduced NCS in myopes, it is hypothesized that NCS is increased in myopes due to an opsin mutation^26^. Such an increase in NCS could also not be observed in this study, but the participants were not tested for this mutation. In general, the abundance of any significant difference in NCS between adult emmetropes and myopes makes it difficult to draw any conclusion regarding the mechanism of myopia progression. Thus, future studies need to test NCS with children to assess whether NCS is altered during myopia progression in the developing eye and could serve for example as a biomarker.

Hypothetically, the NCS measurements, especially for high SFs could have been affected by adaptation to blur, due to the unusual aberration-free stimulus projection, resulting in an underestimation of the true NCS^52,53^. For example, in a previous study, a 15 s adaptation time to a grating stimulus that was 0.75 log10 units above threshold led to a threshold increase of contrast sensitivity of 0.2 log10 units^54^. With an average number of 32 trials tested in the current study, the total summed up stimulus duration would be 16 s. However, the individual stimulus presentation duration of 500 msec was extremely short and not likely to cause adaptation. Additionally, the stimulus presentation was followed by a period of at least 1 to 2 s without a stimulus, with only the background, depending on the response time. Furthermore, because of the implementation of QUEST to determine the individual’s threshold, most of the stimuli were displayed with a contrast close to the threshold, additionally minimizing a influence of adaptation. Given these procedures, any visual adaptation to the stimulus which could have led to an underestimation of the NCS can be neglected.

It was recently shown that for a resolution task with AO correction, visual acuity is correlated to the retina’s Nyquist limit^55^. However, the estimated cut-off frequency from a rational fit exceeded the Nyquist limit of a healthy retina in the current study (see Fig. 3). For a detection task, which was used here, this observation has been reported earlier^23,56^ and, could be explained by aliasing, which allows stimulus discrimination even at SFs beyond the Nyquist limit^23,57,58^. However, central vision contrast thresholds between a resolution and detection task are similar up to a SF of 50 cpd^59,60^meaning that the NCS measured here for SFs between 3 and 36 cpd are not likely affected by aliasing.

Under these circumstances, it is surprising that for the albinism patient, a steep drop in NCS beyond 12 cpd was observed. In this patient, the estimated cut-off frequency was much lower than the retina’s Nyquist limit (about 27 cpd compared to 47 cpd). A related observation was reported earlier for albinism patients, with visual acuity not reaching the Nyquist limit^61^. A possible explanation for this is that in the foveal region of a healthy retina, each cone connects with a single midget retinal ganglion cell, and it was demonstrated that central acuity is directly correlated with the Nyquist limit of the foveolar cone mosaic^55,62^. It is assumed that in albinism the absence of a true fovea goes along with a lack of a preserved private line pathway^61^ resulting in the cut-off frequency and resolution limit being limited by the midget ganglion cell sampling like it was shown in the parafovea of healthy participants^62^.

However, the estimated cut-off frequency matched the visual acuity determined via FrACT closely, while these two read-outs were higher than the letter chart and an OCT-based visual acuity estimation. Due to the underlying algorithm of FrACT, placing the stimulus always at the estimated threshold, it was reported earlier that FrACT visual acuity values are higher on average compared to the traditionally used letter chart^63,64^. Considering the findings for NCS reported here, the FrACT values seem more closely related to the true resolving capability of the individual retina, especially in albinism.

When the pooled data set was divided into two subgroups (22 ± 2 and 32 ± 4 years old), to test age-related influences, the NCS functions of the two groups were significantly equivalent. This is contradictory to the previous finding that the NCS deteriorates with each decade^65^but confirms another study, where only a small difference in NCS functions between younger and elderly participants (average age 21 and 68 years) were observed^66^. Thus, further research is needed to specifically investigate NCS in the healthy aging eye.

In conclusion, the interferometric system used here utilizing a spatial light modulator enabled the measurement of NCS in 48 naïve participants. Robust NCS functions were observed, unaffected by myopia and under-correction. Thus, it can be concluded that long-term under-corrected myopes will be able to achieve normal visual acuity as soon as their refractive errors are sufficiently corrected. Because of the improved optical design and shorter wavelength compared to classic interferometer-based NCS measurements, the currently reported absolute NCS values are slightly higher, but in accordance with previous findings. Therefore, a generally applicable NCS function for young healthy eyes is proposed. For two healthy participants the estimated cut-off frequency is close to the Nyquist limit and higher than the best corrected visual acuity, confirming that human central vision in the healthy eye is mainly limited by the eye’s optics. A first comparison of the normal NCS function with NCS values obtained from an albinism patient revealed a significant NCS degradation. With the estimated cut-off frequency being significantly lower than the Nyquist limit, this leads to the conclusion that vision in albinism is mainly limited by neurological factors. However, more research is needed to investigate this hypothesis in more detail.

## Methods

### Study participants and group assignment

The study adhered to the tenets of the Declaration of Helsinki and was approved by the by the Medicine Faculty Human Research Ethics Committee from the University of Tübingen (616/2022BO2 and 094/2024BO1). Before data collection, the experiment was explained in detail to the participants, and written informed consent was collected from each participant. All data were pseudonymized and stored in full compliance with the principles of the Data Protection Act GDPR 2016/679 of the European Union.

To identify and recruit myopic under-corrected eyes into our cross-sectional observational study, the following recruitment strategy was performed. For a few days, an information booth was installed at a central premise of the Tübingen University. Student passers-by were invited to take part in a quick and free acuity assessment. Those who improved by at least one line on the acuity chart when using negative trial lenses (− 1 D or − 2 D) were encouraged to sign up for our study and get checked in more detail. A total of 48 healthy participants with no known eye diseases (27 females; 27 ± 6 years) were recruited. Additionally, one otherwise healthy Albinism patient (f; 31 years; phenotype: OCA1B; genotype: *TYR* exon 3–5 deletion, autosomal recessive) with known foveal hypoplasia was recruited.

At the first visit, each participant underwent non-cycloplegic objective refraction (iProfiler plus, Carl Zeiss Vision GmbH, Aalen, Germany) and visual acuity testing with their current glasses, if available. Participants were assigned to the emmetropic study group if the respective spherical equivalent (SE) of the objective refraction was ≥ − 0.5 D and ≤ + 0.5 D (a definition widely used in myopia studies^27^ and they achieved a visual acuity of − 0.2 logMAR at 4.5 m distance (VisuScreen 500, Carl Zeiss Vision GmbH, Aalen, Germany). Myopic participants (SE < − 0.5 D) additionally underwent subjective refraction to determine if the participant belonged to the well-corrected or under-corrected group. Subjective refraction was done using a phoropter (ZEISS Visuphor 500, Carl Zeiss Vision GmbH, Aalen, Germany) and a screen (ZEISS Visuscreen, Carl Zeiss Vision GmbH, Aalen, Germany) at 4.5 m distance showing logMAR chart letters. Participants were assigned to the under-corrected group if subjective refraction revealed a deviation of at least 0.5 D in sphere between worn and needed refractive aid. This threshold of 0.5 D refers to the statement that the minimum significant shift in refractive status is a difference of at least 0.5 D^67^. Group assignment was based on the dominant eye, which was then tested during the NCS assessment. Eye dominance was determined with the Miles test: participants were asked to form a triangle with the hands at arm’s length and look through the triangle with both eyes open at a distant target. The eye seeing through the triangle was reported as the dominant eye. All participants who already possessed spectacles (which are mainly the well-corrected myopes) had to assure that they are wearing these for at least 8 h per day and not only for certain activities like driving to be included in the study.

Axial length of the tested eye was measured (IOLMaster 700, Carl Zeiss Meditec, Dublin, CA, USA), and to ensure retinal integrity, foveolar optical coherence tomography (OCT; ZEISS PlexElite 9000, Carl Zeiss Meditec, Dublin, CA, USA) scans were recorded for each participant.

### Interferometer setup

**Fig. 4:**
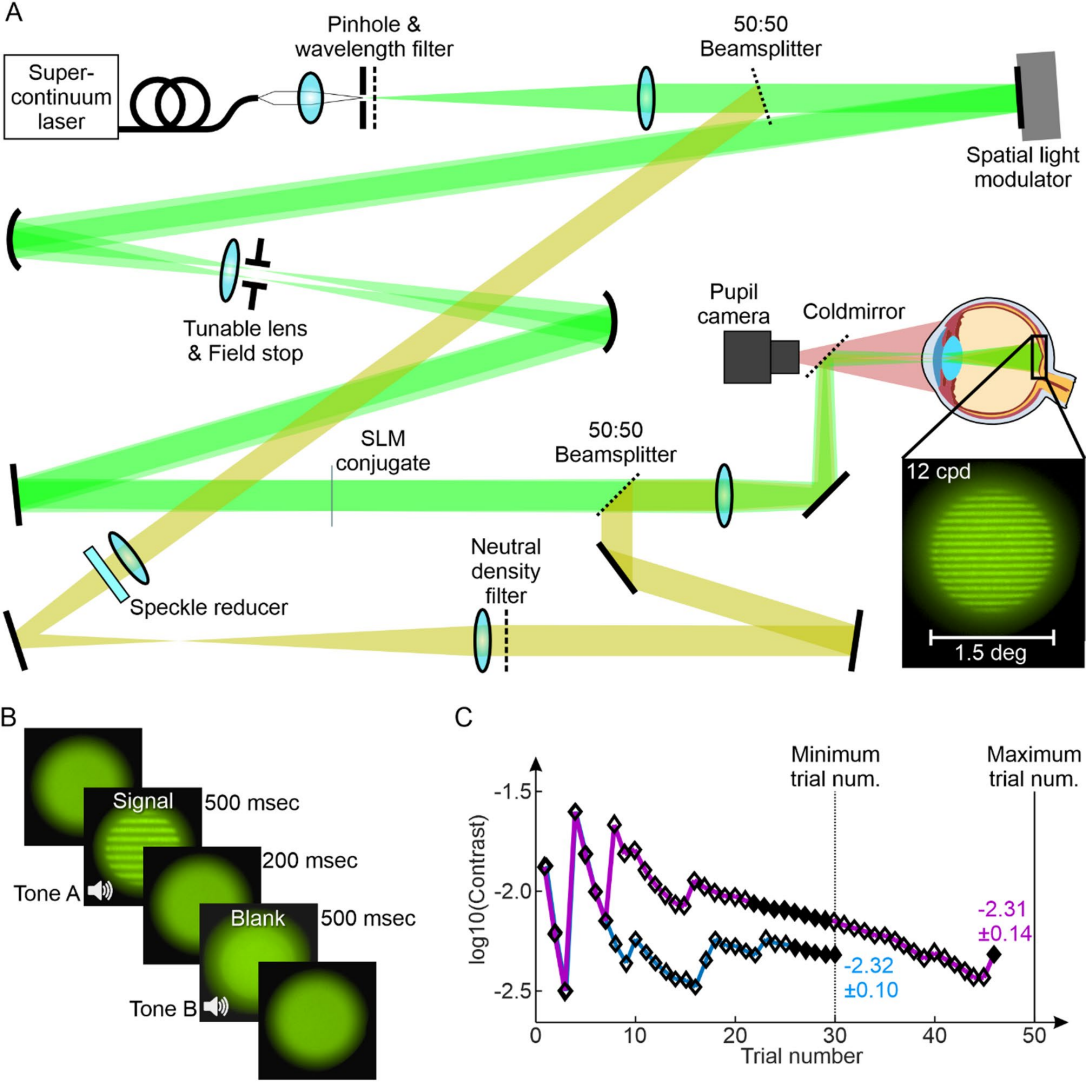
The interferometric setup and psychophysical procedure for the neural contrast sensitivity assessment. (**A**) Schematic beam path of the optical system forming the interference fringes on the retina by using a spatial light modulator (SLM). A motorized aperture adjusts the diameter of the field stop blocking higher order maxima as a result of the SLM based approach splitting the light source into two via a phase mask. The background consisted of the same light spectrum as the stimulus (indicated here with a yellowish path) and was used for a better contrast control as the background was always visible with the stimulus being projected on top of the background light. The tunable lens was adjusted by each participant to have the two spots perfectly overlapping on the retina. An on-axis pupil camera was used to ensure optimal stimulus delivery throughout the experiment. (**B**) Each trial consisted of two intervals and was started by the participant. One interval contained the stimulus, the other one a blank and the participant had to report the stimulus (signal) interval via a button press (2-Interval forced choice). The two intervals were accompanied by two tones and separated by an inter stimulus interval of 200 ms. (**C**) Exemplary experiment progression with QUEST for two runs. Stop criteria were a confidence interval for the current threshold estimate smaller than 0.15 (indicated by filled markers) and a minimum number of 30 trials. If the first criterion was not met after the first 30 trials, additional trials (up to 50 in total) were presented until a SD < 0.15 was reached. Runs not meeting the criterion after 50 trials were repeated.

To investigate the neural contrast sensitivity of the different groups, the threshold of contrast vision was measured psychophysically using a liquid-crystal-on-silicon spatial-light-modulator -based interferometer (Fig. 4A) as described earlier^68^. In brief, the necessary spatially coherent light in this setup was provided by a supercontinuum laser (SuperK Compact, NKT Photonics, Birkerød, Denmark) and the stimulus light (550 ± 5 nm; FBH550-10, Thorlabs GmbH, Bergkirchen, Germany) was filtered from its broad spectrum. The key element of the system, the spatial light modulator (PLUTO-2-VIS-016, Holoeye, Berlin, Germany), was placed in the Fourier plane of the first collimating lens. To create the two laterally separated coherent wavefronts the spatial light modulator displayed two blazed gratings, with each grating providing a tilt that controlled the lateral shift of the wavefront in the image plane of the system. A spherical mirror focused the two beams in the system’s intermediate image plane, where a motorized iris diaphragm (8MID10-40, Standa Ltd., Vilnius, Lithuania) was placed as a field stop to filter out zeroth and higher diffraction orders. Additionally, a tunable lens (EL-12-30-TC, Optotune Switzerland AG, Dietikon, Switzerland) was placed at this position to compensate for the doubling of the field stop in cases of uncorrected refractive error resulting in non-overlapping beams on the retina^40^. The power of the tunable lens was set individually by each participant at the beginning of the experiment to completely overlap the two spots on the retina. After collimating the beam again, the wavefront was focused together with a non-coherent background beam in the pupil plane. The resulting Maxwellian field of view was 1.5° for the stimulus and 1.6° for the background. The non-coherent background was necessary to decrease the available contrast values into the range of the expected thresholds and to reduce masking effects of coherent spatial noise due to the monochromatic light. Spatial coherence of the background was broken via a rotating diffuser foil. The incoherent background light accounted for 90% of the total retinal illuminance^30,69^, which was about 300 Td for the combined field^30,70^. All stimulus parameters were chosen to match the conditions reported in Williams 1985 for better comparability^23,30^.

Because even small head movements would move the pupil out of the beam, the participants were asked to bite on an individually produced bite bar during the measurements. The eye’s pupil was then conjugated with the system’s pupil position via a x-y-z-translation stage. Pupil centration was monitored in real-time throughout the experiment by an on-axis CMOS camera (DMK 27AUP031, The Imaging Source, Bremen, Germany) behind a cold mirror (FM203, Thorlabs GmbH, Bergkirchen, Germany). During the NCS measurement, only the dominant eye was tested while the fellow eye was occluded using an eye patch.

### Psychophysical measurement of the neural contrast sensitivity

The NCS measurement procedure was conducted only for the dominant eye via QUEST (Quick estimate by sequential testing)^71^an adaptive staircase method for threshold estimation, in a two-interval forced choice environment (Fig. 4B) using MATLAB (The MathWorks, Inc., Natick, USA) and the psychophysics toolbox^72^. The participant started each trial individually. After a delay of 300 ms, the first of the two intervals was presented for 500 ms, followed by an interstimulus interval of 200 ms and the second interval (again 500 ms). With the background always visible, the two intervals were projected on top of it accompanied by audio cues helping the participant to separate the two intervals. Signal and blank were assigned randomly into the two intervals. The participant named the interval in which the grating had been perceived via a button press on a controller. Depending on whether the answer being correct or incorrect, the contrast of the next stimulus changed as specified by QUEST. Every 5th trial was either a “catch” trial, displaying a blank in both intervals or a “lapse” trial with maximum contrast in turn. For each run, the participant had to do at least 30 trials. If QUEST´s confidence interval for the current threshold estimate was smaller than 0.15, the run was finished, otherwise more trials, up to a maximum number of 50 trials, had to be done until the before mentioned criterion was met (see Fig. 4C). If the stop criterion was not met after 50 trials the run was terminated and had to be repeated. On average runs were completed with 31.78 ± 3.64 trials, with 95% of trials being completed after 39 trials.

Participants had to do two training runs at the beginning of the experiment with 12 and 24 cpd to familiarize themselves with the unusual stimulus presentation and experiment procedure. Even though normal eye blinking was approved and emphasized, the extensive visual attention during the experiment could have led to a transient “dry eye” sensation. In these cases, artificial tear fluid was offered for self-administration.

In general, a total of seven different spatial frequencies (SF) were measured across three runs, which include 3, 6, 12, 18, 24, 30 and 36 cpd. A subgroup of emmetropes (*N* = 8) was tested additionally at 9 cpd for a more detailed sampling of the NCSF. SFs were tested in pseudo-randomized order to minimize bias from training effects or fatigue. After completion of a set of SFs, a short break (about 5 min) was taken. Each SF was tested three times. If the difference between the lowest and highest threshold was greater than 0.2, a fourth and, if needed, a fifth run for this SF was recorded. This limit of 0.2 was determined empirically during pilot testing based on the observation that repeated measurements at the same SF fluctuate with about ± 0.1. Fluctuation of 0.2 and higher could only be observed during training runs or the participant being inattentive. The final NCS reported here at a given SF for each participant, was calculated as the median of the three closest recordings.

### Adaptive optics retinal imaging and foveolar cone mosaic analysis

Three participants were selected for AO imaging: One emmetropic, one well-corrected myope and the albinism patient. The two healthy participants were selected because they showed a large difference in NCS at a spatial frequency of 36 cpd and AO imaging was intended to resolve the foveolar cone mosaic as a possible explanation for the observed variance. The central ± 150 μm in the dominant eyes was imaged using near-infrared light for imaging and wavefront sensing, filtered dichroically (788 ± 12 nm; FF01-788/12–25, Semrock, Rochester, NY, USA) from the output of a supercontinuum laser light source (SuperK EXTREME, NKT Photonics, Birkerød, Denmark). Adaptive optics correction, run in a closed loop at about 25 Hz, consisted of a Shack–Hartmann wavefront sensor (SHSCam AR-S-150-GE; Optocraft GmbH, Erlangen, Germany) and a 97-actuator deformable mirror (DM97-08; ALPAO, Montbonnot-Saint-Martin, France) placed at a pupil conjugate. The imaging raster spanned a square field of 0.85 × 0.85 degrees of visual angle. The light reflected from the retina was detected in a photomultiplier tube (PMT, H4711-50, Hamamatsu, Japan), located behind a confocal pinhole (0.5 Airy disk diameter). PMT signals were sampled by a field programmable gate array board (ML506; Xilinx, San Jose, CA, USA), producing video frames with 512 × 512 pixels (spatial resolution, 0.1 arcmin of visual angle per pixel) at about 27–30 Hz. To ensure optimal image quality during recording, the pupil’s position relative to the adaptive optics scanning laser ophthalmoscope (AOSLO) beam was carefully maintained^73^. Videos were recorded centered at the preferred retinal locus of fixation. Optimal image quality was found by selecting the best video from five to ten videos recorded using different defocus settings of the deformable mirror. All videos were 10 s long. Acquired AOSLO video frames were spatially stabilized by offline, strip-wise image registration using a modified version of previously published software in Matlab^74^.

The processing pipeline to determine the cone density centroid was described earlier^50^. In brief, the cone center locations in the final montage were labeled in a semi-manual process by a single trained image grader (JA): first, a convolutional neural network was used to annotate retinal images automatically^75,76^ and in a second step manually corrected using custom software in Matlab. Based on the labeled cone center locations, a Voronoi tessellation was computed (Matlab: delaunayTriangulation, voronoiDiagram and voronoin). Each cone was regarded as occupying the space of each corresponding Voronoi cell. Angular cone density (cones/deg^2^) was computed at each image pixel by averaging the combined Voronoi area of the nearest 150 encircled cones around that pixel. Finally, the cone density centroid was determined as the weighted centroid (Matlab: regionprops (‘WeightedCentroid’)) of the highest 20% of cone density values. The participant’s Nyquist limit in term of cut-off frequency was calculated as $\:\nu\:=\sqrt{CDC\_density}/\sqrt{3}$^77^.

### Visual acuity testing and estimation

For a more direct assessment of the best corrected minimum angle of resolution, the two for AO imaging selected participants and the albinism patient underwent visual acuity testing with the Freiburg visual acuity test (FrACT) using the Landolt C in 8 orientations^78^. The screen was placed at a 3.5 m distance and calibrated according to the instructions. The final value reported here for the FrACT visual acuity was the average of three consecutive measurements.

To estimate the albinism patient’s visual acuity based on retinal anatomy, the approach and equation proposed by Woerz et al., 2024 were utilized^31^. To that end, spectral-domain OCT images of the fovea were recorded using a prototype high-resolution device (High-Res OCT, Heidelberg Engineering GmbH, Heidelberg, Germany) with an axial resolution of about 2 μm (in air). The required band ratios were evaluated in the foveolar B-Scan defined by the B-Scan showing the deepest foveal indentation.

### Quantification and statistical analysis

All numerical and statistical analysis was performed in Matlab. The area under the curve was calculated as the numerical integration (Matlab: trapz). Normal distribution was tested via Kolmogorov-Smirnov test (Matlab: kstest). Because data was not normally distributed, the three groups were compared statistically with Wilcoxon’s rank sum test (Matlab: ranksum), after confirmation of homoscedasticity via Brown–Forsythe test for equality of variances (Matlab: vartestn (‘BrownForsythe’)). Additionally, to specifically test for similarity of the found NCS functions, an equivalence test based on the two one-sided tests approach (TOST) was implemented using the Mann–Whitney U-test test^28,29^. Correlations between NCS and the eye’s refractive state were calculated based on the F-test (Matlab: regress) and confidence intervals computed using the functions: fitlm and predict. The violin plots were created with Holger Hoffmann’s Matlab function^79^.

## Supplementary Information

Below is the link to the electronic supplementary material.

Supplementary Material 1

Supplementary Material 2

## Data Availability

The datasets generated during the current study are available from the corresponding author on reasonable request.
