# Supplementary material for: Neural contrast sensitivity is not affected by myopic blur
Source: Sci Rep. 2025 Aug 20;15:30646. doi: 10.1038/s41598-025-15911-y (PMC12368043; doi:10.1038/s41598-025-15911-y)
Supplement: Supplementary file 1 — Supplementary Material 1 [file 41598_2025_15911_MOESM1_ESM.docx]

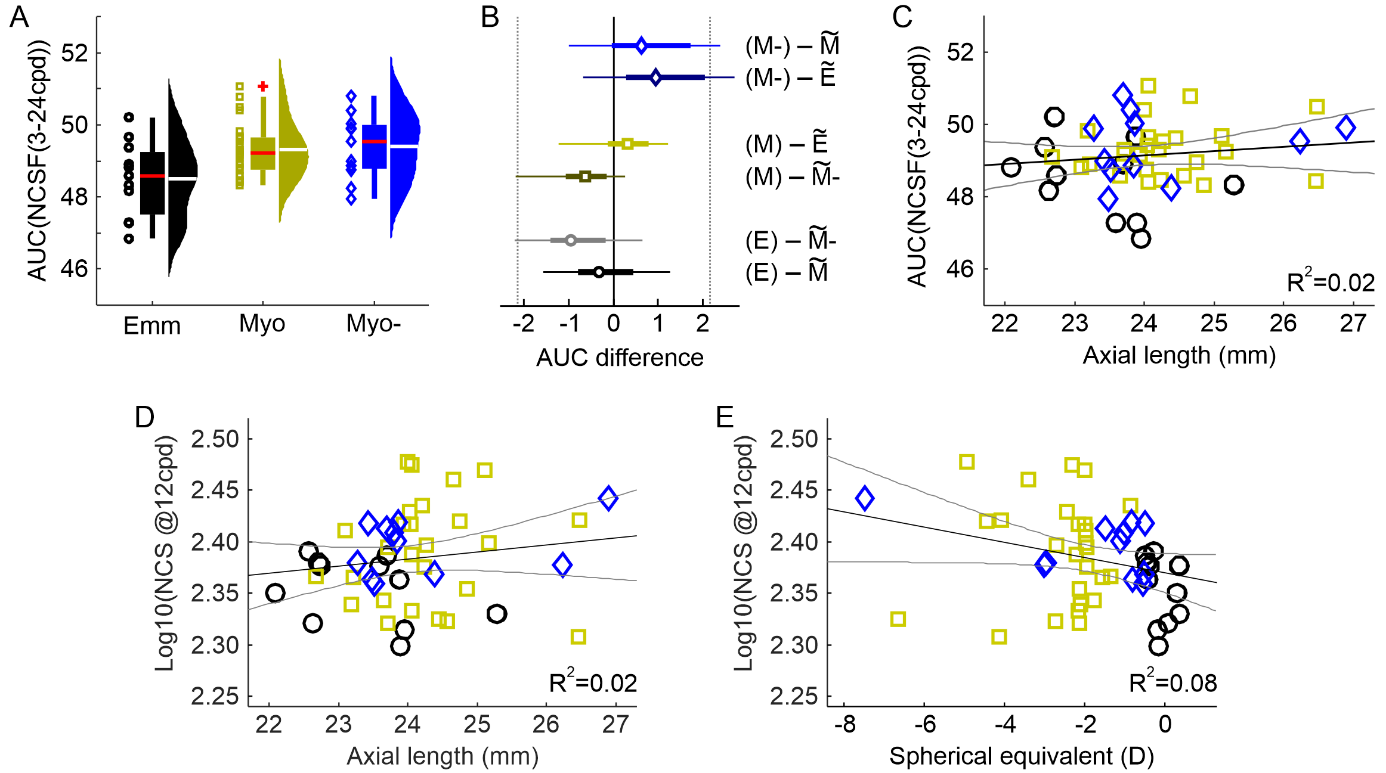


Figure S2: Additional analysis of neural contrast sensitivity functions focusing on the range (A-C) or spatial frequency (D & E) with least variability. A) Area under the NCS curve from 3 to 24 cpd. White lines indicate the mean, red lines the median value. Differences between groups are not significant (Mann-Whitney U-test, all p > 0.05). B) Test for equivalence using the average IQR per tested SF as lower and upper limit (l1 = -2.15; l2 = 2.15) reveals a significant equivalence between AUCs of the three groups (TOST, all p ≤ 0.01). The thick horizontal lines display the group’s interquartile range (25% - 75%) with the thin lines representing the whiskers. The group’s median is given by the marker. Emmetrope: Emm, E; Myope (well): Myo, M; Myope (under): Myo-, M-; X ̃=group median. C) Correlation between AUC (from 3 to 24 cpd) and axial length. D) and E) Correlation between neural contrast sensitivity at 12 cpd and axial length, or, spherical equivalent of objective refraction. All correlations are not significant (p>0.05). Black/Circles = emmetropes; Yellow/Squares = well-corrected myopes; Blue/Diamonds = under-corrected myopes.
