# Supplementary material for: Neural contrast sensitivity is not affected by myopic blur
Source: Sci Rep. 2025 Aug 20;15:30646. doi: 10.1038/s41598-025-15911-y (PMC12368043; doi:10.1038/s41598-025-15911-y)
Supplement: Supplementary file 2 — Supplementary Material 2 [file 41598_2025_15911_MOESM2_ESM.docx]

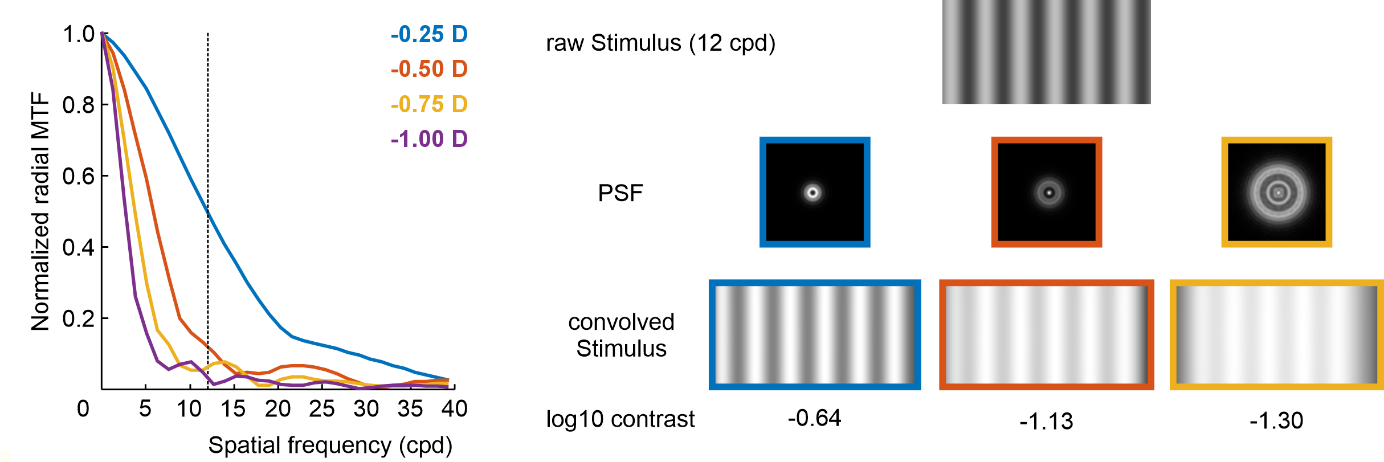


Figure 1: Comparison of the modulation transfer function (MTF) from 0.25 D to 1.00 D under-correction (left side) and its calculated impact on the contrast of an exemplary stimulus with 12 cpd (right side). The retinal stimulus is the result of a convolution of the raw stimulus with the according point spread function considering 0.25 D, 0.50 D, and 0.75 D defocus (all other aberrations set to zero).
